# Long-Term Species, Sexual and Individual Variations in Foraging Strategies of Fur Seals Revealed by Stable Isotopes in Whiskers

**DOI:** 10.1371/journal.pone.0032916

**Published:** 2012-03-14

**Authors:** Laëtitia Kernaléguen, Bernard Cazelles, John P. Y. Arnould, Pierre Richard, Christophe Guinet, Yves Cherel

**Affiliations:** 1 Centre d'Etudes Biologiques de Chizé, Centre National de la Recherche Scientifique, Villiers-en-Bois, France; 2 Centre National de la Recherche Scientifique-Ecole Normale Supérieure, Université Pierre and Marie Curie, Paris, France; 3 School of Life and Environmental Sciences, Deakin University, Burwood, Victoria, Australia; 4 Littoral, Environnement et Sociétés, Centre National de la Recherche Scientifique, Université de La Rochelle, La Rochelle, France; 5 Institut de Recherche pour le Développement, Université Pierre and Marie Curie, Bondy, France; Australian Wildlife Conservancy, Australia

## Abstract

**Background:**

Individual variations in the use of the species niche are an important component of diversity in trophic interactions. A challenge in testing consistency of individual foraging strategy is the repeated collection of information on the same individuals.

**Methodology/Principal Findings:**

The foraging strategies of sympatric fur seals (*Arctocephalus gazella* and *A. tropicalis*) were examined using the stable isotope signature of serially sampled whiskers. Most whiskers exhibited synchronous δ^13^C and δ^15^N oscillations that correspond to the seal annual movements over the long term (up to 8 years). δ^13^C and δ^15^N values were spread over large ranges, with differences between species, sexes and individuals. The main segregating mechanism operates at the spatial scale. Most seals favored foraging in subantarctic waters (where the Crozet Islands are located) where they fed on myctophids. However, *A. gazella* dispersed in the Antarctic Zone and *A. tropicalis* more in the subtropics. Gender differences in annual time budget shape the seal movements. Males that do not perform any parental care exhibited large isotopic oscillations reflecting broad annual migrations, while isotopic values of females confined to a limited foraging range during lactation exhibited smaller changes. Limited inter-individual isotopic variations occurred in female seals and in male *A. tropicalis*. In contrast, male *A. gazella* showed large inter-individual variations, with some males migrating repeatedly to high-Antarctic waters where they fed on krill, thus meaning that individual specialization occurred over years.

**Conclusions/Significance:**

Whisker isotopic signature yields unique long-term information on individual behaviour that integrates the spatial, trophic and temporal dimensions of the ecological niche. The method allows depicting the entire realized niche of the species, including some of its less well-known components such as age-, sex-, individual- and migration-related changes. It highlights intrapopulation heterogeneity in foraging strategies that could have important implications for likely demographic responses to environmental variability.

## Introduction

The concept of ecological niche as an *n*-dimensional hypervolume [1] is a crucial foundation upon which ecologists have tried to understand how species use resources and, thus, the shaping and structuring of communities [2]. Variations in resource exploitation occur along three main dimensions of the ecological niche, namely the spatial, trophic and temporal dimensions [3], [4]. Resource partitioning influences which, and how many species, can coexist and the understanding of resource usage is crucial for explaining species distributions and abundances [5]. Within a population, different age-classes, sexes, morphs and individuals may also specialize at acquiring different resources [6], [7], [8], [9]. Indeed, many generalist populations are composed of individual specialists that use a small subset of the species niche and individual specializations are increasingly recognized as an important component of many ecological and evolutionary processes [9]. Ideally, testing the consistency of individual specialization requires longitudinal sampling in which information is repeatedly collected on the same individuals over the long-term. Practically, however, longitudinal sampling is often labor-intensive, cost-prohibitive and impossible to conduct during the most cryptic stages of an animal's life. Hence, in many cases, inter-individual variability has been ignored or treated as statistical noise, and resource use has primarily been investigated at the population or species level [10].

A recent alternative method is the concept of isotopic niche, since stable isotope analysis provides quantitative information on both habitat (*e.g.* δ^13^C) and resource (*e.g.* δ^15^N) use, factors commonly utilized to define ecological niche space [2], [11]. The isotopic approach is based on the fact that stable isotope ratios in consumer proteins reflect those in diet in a predictable manner. Consumer tissues are stepwise enriched in ^15^N relative to their food and consequently δ^15^N measurements serve as indicators of a consumer's trophic level [12]. In contrast, δ^13^C values vary little along the food chain and are mainly used to determine carbon sources in a trophic network [12] and foraging habitats [13], [14]. Furthermore, the isotopic signature of metabolically inert tissues (*e.g.* keratinous tissues) reflects diet at the time of their growth and, thus, continuously growing tissues record a chronology of movements and dietary history of individuals [15]. Consequently, it was recently suggested that serial sampling of archival tissues can be used as a mean of acquiring high resolution information on the foraging strategies and life histories of individuals, as well as the prevalence of dietary specialization within and among populations [16].

Fur seals are potentially ideal for examining the degree of long-term species-, sexual- and individual-related foraging specialization for several practical and ecological reasons. Firstly, two sibling species, the Antarctic (*Arctocephalus gazella*) and subantarctic (*A. tropicalis*) fur seals breed sympatrically at islands in the Southern Ocean, thus raising the question of the mechanisms allowing their co-existence [17]. Secondly, pinnipeds, including fur seals, show the greatest range of sexual size dimorphism of any higher vertebrate group, which is reflected in different life history strategies of large males and small females [18]. Thirdly, most previous investigations on the food and feeding ecology of fur seals have been conducted on adult females during lactation using the conventional method of food analysis and bio-logging, with almost no information available on the most cryptic sex (the aggressive males that spend only a few weeks on land; [19], [20]) and cryptic life stage (the inter-breeding period during which seals disperse at sea; [21], [22]). Fourthly, species-, sexual- and individual-related foraging differences have already been demonstrated using the isotopic signature of fur seal blood, but blood provides a snapshot that integrates foraging during the last few months preceding sampling with no information over the long-term [23], [24]. Finally, preliminary investigations showed that the isotopic signature of serially-sampled whiskers most likely records the movement pattern of individual fur seals over several consecutive years [25], [26], being thus an unique opportunity to document the foraging strategies of individuals of both sexes all year long.

## Materials and Methods

### Ethics Statement

Animals in this study were cared for in accordance with the guidelines of the ethics committee of the Institut Polaire Français Paul Emile Victor that approved all our fieldwork (Program no. 109, H. Weimerskirch).

### Fieldwork and isotopic analysis

The study was conducted at La Mare aux Elephants (46°22′S, 51°40′E) located on Possession Island, Crozet Archipelago. Antarctic fur seals (*Arctocephalus gazella*) and subantarctic fur seals (*A. tropicalis*) breed sympatrically at this site, with a pup production of 164 Antarctic fur seals and 80 subantarctic fur seals during the study period [27]. Randomly selected breeding males and lactating females of unknown age were captured during December 2001 and January 2002, respectively. Male seals were sedated by intramuscular injection of a tiletamine-zolazepam mixture (Zoletil), while females were captured using a hoop net and placed on a restraint board. From each individual, a single whisker was collected by cutting with a pair of scissors as close to the skin as possible.

Prior to isotopic analysis, whiskers were hand-washed in 100% ethanol and then cleaned in distilled water for 5 min in an ultrasonic bath. Whiskers were measured, dried and cut into 3 mm-long consecutive sections starting from the proximal (facial) end. Sections were weighed on a microbalance within a range (0.03–2.06 mg) that produced meaningful isotopic measurements. Samples were then packed in tin containers, and carbon and nitrogen isotope ratios were determined by a continuous flow mass spectrometer (Thermo Scientific, Delta V Advantage) coupled to an elemental analyser (Thermo Scientific, Flash EA 1112). Results are presented in the conventional δ notation relative to PeeDee belemnite marine fossil limestone and atmospheric N_2_ for δ^13^C and δ^15^N, respectively. Replicate measurements of internal laboratory standards (acetanilide) indicate measurement errors of <0.15‰ for both δ^13^C and δ^15^N. Isotopic data on male *A. gazella* whiskers, previously reported in [26]), have been incorporated in the present study to investigate intra-and inter-specific variations in fur seal foraging strategies.

Five additional lactating female *A. gazella* were sampled at Bird Island (South Georgia) in 1992 as part of other studies [28]. They were aged by counting growth layers of tooth dentin. Seal whiskers were cut into 5 mm long consecutive sections from the root. Isotopic analyses were performed in the laboratory of Donald Schell at Fairbanks (Alaska) in 1994.

Keratinous tissues (including whiskers) are approximately 3‰ ^13^C enriched in pinnipeds compared to their diet [29]. Taking into account both the keratinous effect and the latitudinal gradient in blood δ^13^C values of top predators in the Southern Ocean [14], [30], the isotopic positions of the polar front (PF) and the subtropical front (STF) for fur seal whiskers were estimated at approximately −19 and −16‰, respectively. The subtropical zone (STZ) is defined as the area north of the STF, the subantarctic zone (SAZ, where the Crozet Islands are located) as the area between the STF and the PF, and the Antarctic zone (AZ) as the area south of PF.

### Comparison of the isotopic signature among species, sex and individual seals

Linear mixed-effect models were used to examine the influence of species, sex (tested as fixed effects) and individual identity (random effect) on whisker isotopic signature. Preliminary analyses indicated both δ^13^C and δ^15^N values were time-correlated and exhibit regular oscillation patterns along whisker length. Consequently, the isotopic signature of each whisker was modeled by a sinusoidal function, which is a sum of a sine and a cosine. The effect of the explicative variables on (i) the mean isotopic value [that corresponds to the intercept], as well as on (ii) the amplitude of oscillations [sine and cosine coefficients] [31] were tested in a single model. The whisker-axis was first converted into a time scale, assuming that the cycles were annual (see Discussion). Variance components were estimated separately for δ^13^C and δ^15^N values and the most appropriate model was selected for each isotopic ratio using the Akaike's Information Criterion (AIC).

Since whiskers were not plucked, their most recently synthesized tissue remained under the skin. Consequently, the time zero (basal proximal section) of each whisker corresponded to different times, depending both on the unknown length of the under skin part of the whiskers and on their growth rates. Isotopic values were, thus, time-synchronized, and the first few proximal (more recent) sections were excluded from the variance analysis. Phase synchronization was performed by doing a cross-correlation analysis for each individual between its time series and a series of reference.

### Time series analysis

Since most whiskers showed δ^13^C and δ^15^N oscillation patterns, time series were analyzed in order to characterize the periodicity of isotopic variations, to determine whether the period of oscillations was constant along the whisker length and if oscillating patterns in δ^13^C and δ^15^N values were synchronized. Analyzing the frequency composition of time series is classically achieved by using Fourier series. Frequencies that contribute the most to the variance of the series are identified and periodicity, if present, is detected. However, Fourier analysis requires the assumption of stationary time series and it is not able to characterize changes in frequency through time. In contrast, wavelet analyses enable the variability of a time series in both the time domain and the frequency domain to be described by performing a local time-scale decomposition of the signal. The spectral characteristics of the series can then be estimated as a function of time [32], [33].

**Figure 1:**
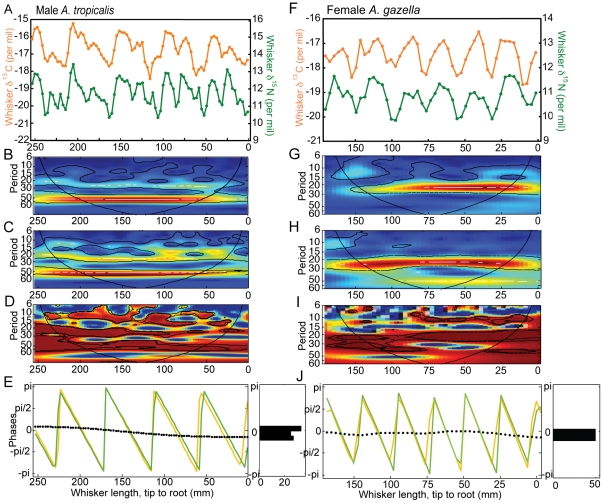
Wavelet and phase analyses of δ^13^C and δ^15^N values of serially sampled whiskers. (A) and (B) refer to two representative fur seals, one male *A. tropicalis* and one female *A. gazella*, respectively. The wavelet spectra decomposes the variance of time series over time (x axis) and frequencies (y axis), allowing the detection of any variation in the periodicity of the fluctuating patterns along the length of the whisker. The color gradient, from dark blue to dark red, codes for low- to high-power values. The hair black line represents the 5% significance levels computed based on 500 Markov bootstrapped series. The black contour indicates the cone of influence that delimits the region not affected by edge effects. (a) Whisker δ^13^C (orange) and δ^15^N (green) time series. The proximal end (time zero), i.e. the youngest part of the whisker appears on the right of plots. (b) and (c) Wavelet power spectrum of the δ^13^C and δ^15^N time series, respectively. The tested frequencies ranged from 6 mm to 50% of whisker length. (d) Wavelet coherency between δ^13^C and δ^15^N time series. (e) δ^13^C (black) and δ^15^N (red) phase analysis (large left panel) and distribution of the phase difference between the two isotopic ratios (small right panel). Phase analyses were performed for lengths ranging between 50 and 60 mm and between 18 and 28 mm for male *A. tropicalis* and female *A. gazella*, respectively.

The wavelet transform represents the contribution of a set of frequencies ‘
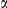
’ to the signal at different time position ‘
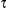
’. It is calculated based on the convolution product between the time series 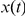 and a wavelet function 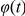 that is dilated/translated onto the signal: 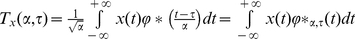, where * denotes the complex conjugate form. The continuous and complex Morlet wavelet function: 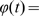 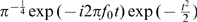 , which allows dissociating easily the phase from the amplitude of the studied signal due to its complex form, was used. The relative importance of frequencies for each time step can be represented in the time/frequency plane (Fig. 1b,c) to form the wavelet power spectrum (WPS) given by: 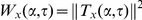 and its average: 
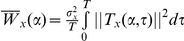
, with 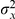 the variance of *x(t)* and *T* the time duration of the series. The wavelet transformed can be viewed as a convolution in a window positioned at time ‘
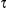
’ with a window width proportional to the frequency ‘
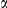
’. Then for a given frequency ‘
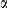
’, the computation of the wavelet transform along the time series needs that the series will be increased by an half window width at the beginning and at the end. This augmentation is done by adding zero to the series that is known as zero padding [32]. The cone of influence on the wavelet graphs delimits the region affected by zero padding. The spectral information within this cone should be interpreted with caution [32], [33]. The 5% significance level of the patterns exhibited by the wavelet approach was determined with bootstrapped simulations, using 500 surrogate data sets based on resampling with a Markov process scheme [34].

The wavelet analysis can be extended to bivariate cases to analyze patterns of covariation between two signals. The cross-spectrum of two series quantifies the local covariance and the wavelet coherency gives an estimation of the linear correlation between the spectra of the two series, at each time position. The cross-spectrum and the square wavelet coherency are defined as: 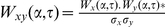 and 
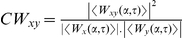
, respectively with 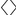 indicating smoothing over time and scales. The square wavelet coherency quantifies the association between two time series but to have the direction of this association we have to compute the phase difference between these time series. With complex wavelets such as the Morlet wavelet, the phase difference is computed based on the imaginary and real parts of the wavelet cross-spectrum as: 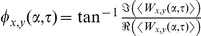. The distribution of the 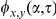 informs us about the direction of the association: a broad and uniform distribution characterizes the lack of phase association between the two time series, while an unimodal distribution of the phase difference indicates there is a preferred value of 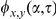 and thus a statistical tendency for the two time series to be phase locked. Then, the quantification of this tendency was tested using the index of generalized entropy [34].

## Results

The number of whisker sections analyzed for each individual fur seal varied from 23 to 111, depending on the length of its whisker (mean 56±19 sections, for a total of 1,953 analyzed samples). Whisker isotopic signatures were spread over a large range, with δ^13^C and δ^15^N values varying from −25.8 to −14.6‰ (an 11.2‰ difference) and from 7.1 to 14.7‰ (7.6‰), respectively (Table 1). Overall, large isotopic differences have been observed between species, sexes and individuals.

**Table 1:** Overall length, δ^13^C and δ^15^N values and C∶N mass ratio of fur seal whiskers from Crozet Islands.

	*n*	Length	Samples	δ^13^C	δ^15^N	C∶N mass ratio
		(mm)	(n)	(‰)	(‰)	
***Arctocephalus gazella***						
Males	10	213±64	710	−19.0±2.7 (−25.3 −14.6)	11.0±1.5 (7.1–14.5)	2.86±0.03
Females	10	146±46	486	−17.5±1.1 (−25.8 −16.1)	10.6±0.6 (8.4–11.9)	2.86±0.04
***Arctocephalus tropicalis***						
Males	5	191±49	317	−16.7±0.8 (−18.6 –14.7)	11.7±0.9 (10.0–14.4)	2.85±0.03
Females	10	133±27	440	−16.8±0.4 (−18.3 −15.7)	10.6±0.8 (8.6–14.7)	2.88±0.02
			426^a^	−16.7±0.4 (−18.2 −15.7)	10.5±0.6 (8.6–12.4)	2.88±0.02

Values are means ± SD, with ranges in parentheses.

a Fourteen samples were deleted because they corresponded to the suckling period of two females when they were pups, thus increasing the δ^15^N range (see text and Fig. 4).

### Isotopic signatures of species, sexes and individuals

**Figure 2:**
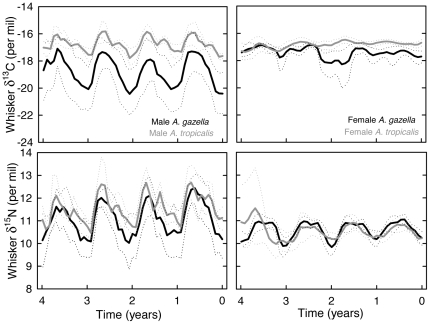
Mean δ^13^C and δ^15^N values (solid lines) and confidence intervals (dotted lines) of fur seal whiskers. Isotopic values were averaged by species (*A. gazella* (black) and *A. tropicalis* (grey)) and sex (males (left panels) and females (right panels)) over a time period of 4 consecutive years. Time zero that corresponds to the newly synthesized part of the whiskers appears on the right of plots.

Whisker δ^13^C values varied between species, with *A. gazella* showing lower values than *A. tropicalis*. Model estimated a 1.9‰ difference between males (means ± the mean amplitude of the cyclic patterns: −18.7±1.2‰ and −16.8±0.8‰ for *A. gazella* and *A. tropicalis*, respectively) and a 0.8‰ difference between females (−17.6±0.4‰ and −16.8±0.1‰, respectively) (Fig. 2). Mean whisker δ^13^C values also varied with sex in *A. gazella* (males had a 1.1‰ lower value than females), but not in *A. tropicalis*. All explanatory variables (species, sex and the two-way interaction) had a significant effect on the amplitude of the δ^13^C oscillations (Fig. 2, Table 2). Male *A. gazella* exhibited the largest δ^13^C oscillations along their whiskers (mean amplitude of 1.2‰) and female *A. tropicalis* the lowest (0.1‰).

**Table 2:** Species and sex effects on whisker isotopic signatures: mean isotopic values [intercept] and mean amplitude of oscillations [√(Sine coefficient^2^ + Cosine coefficient^2^)].

	Intercept		Cosine coefficient		Sine coefficient	
	Value (*P* values)	95% CI	Value (*P* values)	95% CI	Value (*P* values)	95% CI
**δ^13^C model**						
Species	−1.93 (0.002)	[−3.07; −0,79]	−0.33 (0.022)	[−0.61; −0.05]	−0.40 (0.769)	[−0.69; 0.10]
Sex	0.02 (0.191)	[−1,10; 1.14]	0.60 (<0.001)	[0.30; 0.89]	0.17 (<0.001)	[−0.01; 0.34]
Species: Sex	1.08 (0.058)	[−0.37; 2.53]	–	–	0.59 (<0.001)	[0.24; 0.95]
**δ^15^N model**						
Species	0.20 (0.246)	[−0.17; 0.58]	0.18 (0.027)	[0.02; 0.34]	–	–
Sex	0.76 (<0.001)	[0.38; 1.14]	−0.26 (0.019)	[−0.48; −0.04]	−0.34 (0.137)	[−0.54; −0.45]
Species: Sex	–	–	–	–	–	–

Results are given with associated *P* values and 95% confidence intervals. – indicates factors not selected by either δ^13^C or δ^15^N models.

Mean δ^15^N values were explained by sex but not by species, with males showing higher values than females. Model estimated 0.8‰ differences between males and females of both species (means ± the mean amplitude of the cyclic patterns; 11.3±0.8‰ and 10.5±0.5‰ for male and female *A. gazella*, and 11.5±0.7‰ and 10.7±0.3‰ for male and female *A. tropicalis*, respectively) (Fig. 2). Amplitudes of δ^15^N values were also higher in males than in females (Fig. 2, Table 2). No species difference was found between males, but the amplitude of δ^15^N oscillations was significantly higher in female *A. gazella* than *A. tropicalis*.

**Figure 3:**
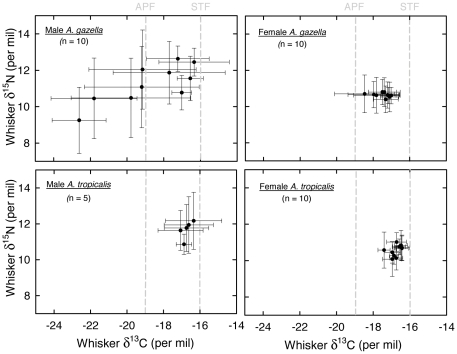
Whisker δ^13^C and δ^15^N values of individual fur seals. Values are means ± the mean amplitude of the cyclic patterns. Note that bars do not represent standard or error bars, but the entire isotopic range of seals. Dashed gray lines illustrate the isotopic estimation of fronts (see text). APF: Antarctic Polar Front; STF: Subtropical Front.

A strong individual effect was found both on δ^13^C and δ^15^N values and on the amplitudes of their oscillations, with the random effect explaining 71 and 54% of the variance of δ^13^C and δ^15^N models, respectively. Inter-individual isotopic differences were mainly observed in male *A. gazella* (Fig. 3). Within that group, mean δ^13^C and δ^15^N individual values ranged from −22.6 to −16.3‰, and from 9.2 to 12.4‰, respectively, and the isotopic amplitudes ranged from 0.6 to 3.3‰, and from 0.7 to 2.2‰, respectively. By contrast, male *A. tropicalis* and females of both species exhibited lower inter-individual variations, with large overlaps of the individual isotopic values within the three groups (Fig. 3).

The δ^13^C estimations of the Subtropical and Polar Fronts at −16‰ and −19‰, respectively, allowed delineating the main latitudinal foraging ranges of fur seals (Table 3). The four groups of fur seals foraged primarily in subantarctic waters, but unlike *A. tropicalis*, *A. gazella* also fed in the Antarctic Zone. Sexual differences were obvious, with females of both species foraging almost exclusively in the Subantarctic Zone, where they occupied a lower trophic position than males. When foraging within the same water masses, males of both species had identical isotopic signatures (Table 3).

**Table 3:** Foraging areas (and the corresponding isotopic signatures) of *Arctocephalus gazella* and *A. tropicalis* according to their whisker δ^13^C values and the estimated isotopic position of the Polar Front (−16‰) and Subtropical Front (−19‰) for whiskers.

			Antarctic Zone				Subantarctic Zone				Subtropical Zone			
Groups	Individuals	Samples	Individuals	Samples	δ^13^C	δ^15^N	Individuals	Samples	δ^13^C	δ^15^N	Individuals	Samples	δ^13^C	δ^15^N
	n	n	n	n (%)	(‰)	(‰)	n	n (%)	(‰)	(‰)	n	n (%)	(‰)	(‰)
***Arctocephalus gazella***														
Males	10	710	8	316 (44.5)	−21.7±1.3	10.0±1.3	9	329 (46.3)	−17.1±0.7	11.7±1.0	6	65 (9.2)	−15.4±0.4	12.8±0.4
Females	10	486	5	31 (6.4)	−20.9±1.6	9.9±0.9	10	455 (93.6)	−17.3±0.5	10.7±0.5	0			
***Arctocephalus tropicalis***														
Males	5	317	0				5	257 (81.1)	−17.0±0.6	11.4±0.7	4	60 (18.9)	−15.5±0.3	12.9±0.5
Females	10	426^a^	0				10	415 (97.4)	−16.8±0.4	10.5±0.6	4	11 (2.6)	−15.9±0.1	11.5±0.4

**Figure 4:**
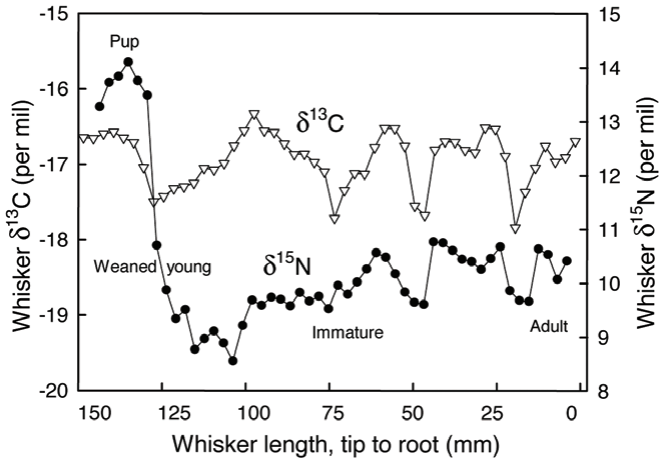
Whisker δ^13^C (open triangles) and δ^15^N (filled circles) values of a female *A. tropicalis*. The proximal end (time zero), i.e. the youngest part of the whisker appears on the right of the plot. The high δ^15^N values followed by an abrupt decline in the oldest part of the whisker most likely represent the suckling period, weaning and subsequent first foraging trip at sea of the young seal (see text).

Values are means ± SD.

a Fourteen samples were deleted because they corresponded to the suckling period of two females when they were pups (see text and Fig. 4).

### Periodicity of isotopic signatures

Most of the males' isotopic signatures exhibited consistent δ^13^C and δ^15^N oscillation patterns all along the length of their whiskers (significant cycles detected on at least 80% of the cone of influence (COI)), whereas isotopic results from females' vibrissae showed more contrasted results (Table 4). In 80% (24/30) of males, δ^13^C and δ^15^N time series showed significant cycles along the whole whisker length, 10% (3/30) exhibited cyclic patterns for only a part of the whiskers (between 50 and 80% of the COI), and 10% (3/30) displayed no oscillation at all. In contrast, 52% (21/40) of the females' time series exhibited significant isotopic cycles all along the whisker length, 10% (4/40) for only a part of their whiskers, but 38% (25/40) presented no oscillations at all. Coherency and phase analyses were performed when both δ^13^C and δ^15^N time series from a given seal were cyclic for at least half of its whisker. In all cases, δ^13^C and δ^15^N values were linearly correlated and synchronous (Fig. 1). Importantly, wavelet analysis showed that periodicity in δ^13^C and δ^15^N values is constant all along the length of a given whisker.

**Table 4:** Periodicity in whisker δ^13^C and δ^15^N time series using wavelet analysis.

	*n*	Cyclic δ^13^C time series	Cyclic δ^15^N time series	Isotopic cycles	Growth rate
		(*n*)	(*n*)	(*n*)	(mm·day^−1^)
***Arctocephalus gazella***					
Males	10	9 (>80% COI)	7 (>80% COI) 2 (>50% COI)	4.2±1.4	0.14±0.02
Females	10	3 (>80% COI) 2 (>50% COI)	10 (>80% COI)	5.0±1.2	0.08±0.02
***Arctocephalus tropicalis***					
Males	5	4 (>80% COI)	4 (>80% COI) 1 (>50% COI)	3.8±0.7	0.14±0.04
Females	10	1 (>80% COI) 1 (>50% COI)	7 (>80% COI) 1 (>50% COI)	4.2±0.9	0.09±0.02

Here we report the number of isotopic time series that exhibited significant cycles either all along the length of the whiskers (at least 80% of the cone of influence (COI), which is the region of the power spectrum that is not affected by the edge effects) or only in a smaller part of the series (between 50 and 80% of the COI). Growth rate was calculated assuming that cycles were annual (see text).

The mean number of cyclic patterns recorded per whisker varied from 3.8 to 5.0 depending on species and sex, with an absolute range of 2.7 to 7.2 cycles per whisker (Table 4). The period of oscillations varied between sexes and individuals, from 35 to 73 mm (mean 51±11 mm) for males, and from 17 to 44 mm (30±7 mm) for females. Assuming that the oscillations were annual (see Discussion), growth rates averaged 0.14 and 0.08 mm·day^−1^, respectively (Table 4), and each 3 mm whisker section corresponded to ∼21 and ∼37 days for males and females, respectively.

Tooth analysis indicated that the five female *A. gazella* from South Georgia were 4–12 years-old at the time of sampling. All their whiskers exhibited isotopic oscillations with a constant periodicity all along their length (data not shown). The number of isotopic cycles ranged from 3.6 to 8.8, depending on individuals. Importantly, the number of isotopic cycles was always ≤ to the age of the animals. Whisker growth rates amounted to 0.06–0.09 mm·day^−1^ (Table 5).

**Table 5:** Length, number of isotopic cycles, and growth rate of whiskers collected from five lactating female *A. gazella* from Bird Island (South Georgia) in 1992.

Individual	Length	Samples	Growth rate	Isotopic cycles	Age	% life span recorded
	(mm)	(*n*)	(mm·day^−1^)	(*n*)	(years)	in whiskers
# 2923	160	29	0.06	7.1	7	100
# 2924	125	24	0.07	4.6	6	77
# 2925	230	45	0.07	8.8	12	73
# 2926	115	23	0.09	3.6	4	90
# 2927	140	27	0.08	4.6	9	51

## Discussion

The isotopic signature of serially sampled keratinous tissues records longitudinal habitat and diet changes over the long-term. The method already highlighted individual diet specialization (vibrissae: [10]; scutes: [35]), seasonal diet variations (hairs: [36], [37]; horns: [38]) and migratory patterns (baleen plates: [39]). To the best of our knowledge, however, the present study is the first to use δ^13^C and δ^15^N values of whiskers to investigate simultaneously species- and sex-related foraging strategies together with inter- and intra-individual variations in the movement patterns of wild mammals. Moreover, the ease and simplicity with which whisker isotopic values can be gathered and analyzed provides a time-window that is difficult or near impossible to record using direct observations and bio-logging.

### Methodological comments

Isotopic analyses of serially sampled whiskers of *Arctocephalus gazella* and *A. tropicalis* showed that changes in δ^13^C and δ^15^N values co-varied. Moreover, δ^13^C and δ^15^N cycles were found in a majority of whiskers, and wavelet analysis indicated that their periodicity is constant all along the length of a given whisker. The record of regular isotopic oscillations per whisker together with a decrease in whisker diameter from the root to the tip are in agreement with otariid whiskers growing and abrading continuously at a constant rate and not being shed [25]. Two consequences arise from this growth pattern. Firstly, unlike phocid whiskers that do not grow at a constant rate and are shed [25], [40], otariid whiskers constitute an ideal recording tissue to reconstruct individuals' isotopic history [26]. Secondly, from a methodological point of view, whiskers must be cut into constant-length sections, so that each section has a similar temporal integration. This allows efficient analytical tools (e.g. wavelet analysis) to be used, and thus a better and easier biological interpretation of isotopic variations with time.

The main caveat in using isotopic records from serial-sampled keratinous tissues is the lack of accurate growth rates, thus precluding knowing with certainty the time frame over which serial tissues sampled reflect ecological information [16]. Consistent isotopic oscillations along whiskers were previously noted in Steller sea lions (*Eumetopias jubatus*). Each oscillation was interpreted as reflecting a complete annual cycle, because the estimated whisker growth rate for each oscillation in wild animals was identical to the range of growth rates in captive individuals [25]. A further and strong argument supporting the annual periodicity of isotopic cycles is that the number of cycles from whiskers of South Georgia fur seals was always equal or lower than their ages (Table 5). Moreover, whisker growth rates of female *A. gazella* from South Georgia were identical to those from Crozet Islands. Hence, a single whisker recorded a substantial part of the animal's life, corresponding to 51–100% of the life span of the five aged seals.

Whisker growth rate was higher in males than in females for both fur seal species. Assuming a constant whisker abrasion rate in both sexes, a higher whisker growth rate in males together with a thicker whisker root is a likely explanation for why male whiskers are longer than those in females (Table 1). This suggests that longer whiskers is a secondary sexual characteristic within the genus *Arctocephalus* facilitated by faster whisker growth. Nonetheless, whiskers of both sexes recorded the same mean number of isotopic cycles with the better temporal resolution in male whiskers resulting from the shorter time integration in each sampled whisker section (∼3 weeks compared to 5–6 weeks in females).

### Biological interpretation of the isotopic oscillations

Both ecological and physiological processes affect the isotopic signature of animals. Many marine mammals experience seasonal cycles in food intake and energy demands that may potentially alter and blur the ecological interpretation of their isotopic signatures [16]. For example, territorial male fur seals fast on land at the beginning of the reproductive cycle [18], and fasting is known to increase δ^15^N values but not δ^13^C values of keratinous tissues [41], [42]. In the same way, gestation, and possibly lactation decreases δ^15^N values with no changes in δ^13^C values of females [43]. Several arguments, however, preclude any major driving effect of nutritional and reproductive physiology on the isotopic signature of fur seal whiskers. Firstly, whisker δ^13^C and δ^15^N values co-varied synchronously. Secondly, whisker isotopic cycles were found in both males and females. Thirdly, isotopic oscillations with the same periodicity occurred in females of both species that present different durations of the lactation period (∼4 and ∼10 months for *A. gazella* and *A. tropicalis*, respectively; [44]). We are thus confident that, when present, the whisker isotopic oscillations mainly reflect annual foraging cycles of male and female fur seals over several consecutive years [25], [26]. This does not preclude, however, potential physiological isotopic changes of lower amplitudes that need to be thoroughly investigated on captive seals.

Two female *A. tropicalis* showed an unusual isotopic pattern marked by typical δ^13^C and δ^15^N oscillations in the most recent part of the whiskers, but not at their tip. Instead the oldest part of the whiskers was characterized by the highest recorded δ^15^N values followed by a sharp δ^15^N decrease. Such high δ^15^N values characterized nursing young that feed on milk and are thus at a higher trophic level than their mothers. While such a δ^15^N suckling effect has been reported in ontogenetic analyses of bones and tooth annuli of marine mammals [16], it has not previously been demonstrated in their whiskers. Whiskers, thus, recorded at a temporally fine scale the complete ontogenetic sequence from birth to the adult age: (i) an extended suckling period, (ii) an abrupt weaning, associated with a rapid δ^15^N drop, (iii) a long transition period during which young seals fed on relatively low trophic level prey, and (iv) the progressive setting up of a more regular foraging strategy over years (Fig. 4).

Whiskers presented isotopic oscillations in most cases, but with significant differences between species and sexes. Almost all males exhibited whisker isotopic cycles of large amplitudes, thus contrasting with female whiskers that showed, when present, oscillations of lower amplitudes. Such variations can be directly related to sexual differences in annual time-budgets of fur seals. Males are present at breeding beaches during a few weeks at the beginning of the reproductive cycle, and, consequently, they may disperse far away from the breeding colonies during most of the year. By contrast, fur seal mothers alternate foraging trips at sea and suckling bouts ashore during the whole lactation period during which they are, thus, confined to a more limited foraging range close to the colony [18]. In the same way, the longer lactation period of *A. tropicalis* is the likely explanation of their higher number of whiskers that did not present isotopic oscillations, and, when present, with lower amplitudes than female *A. gazella*.

### Biological interpretation of the isotopic signatures

The isotopic signature of whiskers confirmed and expanded considerably the species-, sex- and individual-related differences in foraging strategies that were previously depicted by using the isotopic signature of blood on the same individual fur seals [23]. Unlike blood, whiskers represent an archive of feeding ecology over several consecutive years that include the most cryptic life stages of the seals. Hence, whisker data add important information to the movement patterns and inter-breeding foraging grounds of *A. gazella* and *A. tropicalis*. Overall, mean isotopic signatures highlight trophic segregation between fur seals breeding at the Crozet Islands. Whisker δ^13^C and δ^15^N values defined three foraging areas and two trophic levels, respectively, thus allowing characterization of four distinct but overlapping trophic niches, depending both on species and sex.

The results of the present study suggest the main segregating mechanism among the various groups operates at the spatial scale. Using latitudinal variations in δ^13^C values of marine organisms in the Southern Ocean [14], [30], the average isotopic signatures shows a spatial foraging gradient from southern and colder waters to northern and warmer areas in the order: male *A. gazella*, female *A. gazella*, and male and female *A. tropicalis*. This spatial segregation reflects primarily the species breeding range, since *A. gazella* and *A. tropicalis* occur within the Antarctic and Subtropical Zones, respectively, and overlap in the Subantarctic Zone. Noticeably, however, the present study extends the segregation to the whole cycle, with *A. tropicalis* not foraging in Antarctic waters and female *A. gazella* not dispersing in the subtropics (Table 3). Spatial segregation in foraging is, thus, a major mechanism allowing co-existence of closely-related fur seal species [17], as already described for other top predators from the Southern Ocean [45], [46], [47].

Size dimorphism is considered as a main force driving gender differences in the food and feeding ecology of marine mammals [18]. Indeed, the much larger size of males in fur seals increases their physiological diving capacity such that they dive longer and deeper than females [19], [20], [48]. Our isotopic data nevertheless suggest that annual time-budget is a more important factor than size in shaping the male and female foraging strategies. Indeed, the lack of parental care allows males to disperse immediately at the end of the harem period, while females feed dependent young at that time. Parental care, thus, explains why females spent more time in subantarctic waters than males, and the longer duration of the lactation period explains why female *A. tropicalis* spent more time in subantarctic waters than female *A. gazella*.

Another segregating mechanism among genders operates at the trophic scale (δ^15^N), with males feeding at a higher trophic position than females. The diet of males at Crozet Islands is unknown but lactating females of both species feed almost exclusively on myctophid fish [23]. This, together with the known diet of males elsewhere [49] and the 1‰ δ^15^N gender difference (i.e. ∼1/3 of a trophic level) observed in the present study, suggests that males prey upon myctophids but that they also consume a significant amount of higher trophic level prey, most likely larger fish or oceanic squids. Regardless of the exact prey of males the sexual dietary difference is another mechanism limiting intra-specific competition for resources.

In contrast, males and females of the two species potentially competed for the same prey when they foraged within the same water masses. For example, male *A. gazella* and *A. tropicalis* occupied the same trophic positions in both subantarctic and subtropical waters. Thus, inter-specific differences in trophic niche seem smaller than inter-sexual differences, suggesting that inter-sexual differences in trophic ecology actually evolved before the recent speciation of the two taxa [50].

Inter-individual variations were low within three (male *A. tropicalis* and females of both species) of the four investigated groups of fur seals, with great overlaps in their mean whisker isotopic values and amplitudes. In contrast, male *A. gazella* showed large inter-individual isotopic variations indicating that individual specialization occurred over the long-term. Two complementary mechanisms may have allowed partial or complete individual segregation. Firstly, different mean isotopic values indicate that males *A. gazella* fed in different habitats and on different prey. Secondly, different amplitudes of isotopic oscillations show that some males performed large latitudinal migrations (over thousands of kilometers), while others remained within the same water masses year round. Indeed, the foraging cycle of one individual encompassed three oceanographic zones, from Antarctica to the subtropics, while two and one male foraged exclusively within the Antarctic and Subantarctic Zones, respectively. Four male *A. gazella* had repeatedly very low whisker δ^13^C values that were always associated with very low δ^15^N values. Those individuals migrated south in high-Antarctic waters where they most likely fed on Antarctic krill *Euphausia superba* [26]. Some male *A. gazella*, therefore, used the same strategy as subantarctic seabirds that forage in Antarctica to molt at the end of summer and autumn, when krill is plentiful in ice-free areas [47], [51].

Finally, one of the most remarkable findings of the present study is the consistency of values and amplitudes of isotopic oscillations within most of the whiskers. Constant and reproducible intra-individual isotopic cycles indicate that many individuals followed the same movement pattern year after year. For example, some male *A. gazella* alternate between feeding on krill in high-Antarctica and feeding on myctophids in northern waters over 5–7 consecutive years. Other seals showed progressive changes from year to year, with whiskers of two females depicting the progressive setting up of their foraging strategies (Fig. 4). Individual fidelity to wintering areas of pinnipeds and seabirds has previously been observed by both direct tracking and the stable isotope method, but generally for no more than 2–3 consecutive years [52], [53], [54], [55]. Unlike those investigations, the primary advantage of the isotopic signature of whiskers is that repetitive captures of the same marked individuals are not necessary and that one capture provides information on an average of 4–5 (up to 8) complete annual cycles.

### Conclusions

Evaluation of species niche provides valuable insight into a species' role within communities and ecosystems [1], and evaluation of intrapopulation niche variation has important ecological, evolutionary and conservation implications [9]. The present study clearly illustrates the usefulness of stable isotopes as niche indicators because whisker isotopic signatures yield unique long-term information that integrates the three dimensions (spatial, trophic and temporal) of the ecological niche. The method is based on the collection of information at the individual level and it may record the life of animals from birth to death. It therefore depicts the entire realized niche of the species by taking into account some of its less well known components such as age-, sex-, individual- and migration-related changes. The large degree of variation we observed highlights the complexity of the ecological structure of animal population and suggests that extrapolating species-level processes from a small subset of individuals is inappropriate in many species, including pinnipeds [56]. The isotopic method points out different ecological structure of fur seal populations in relation to the use of the species niche. The population of *A. tropicalis* is divided into two sex-related groups that show limited intra-group variations and small overlap in their use of the species niche. The ecological structure of the population of *A. gazella* is different. Females present small between-individual variations and they use a small niche subset. By contrast, males overall use the entire species niche, including the females' habitat and diet, and they present large between-individual variations, each male using a small to large subset of the niche width. Such striking heterogeneity in foraging strategies could have important consequences in terms of life-history traits and evolutionary fitness and thus implications for likely demographic responses to environmental variability.
